# Parliament’s Constitution: Legislative Disruption of Implied Repeal

**DOI:** 10.1093/ojls/gqad004

**Published:** 2023-03-13

**Authors:** Asif Hameed

**Affiliations:** Lecturer in Law, Royal Holloway, University of London

**Keywords:** constitution, legislation, implied repeal, priority, parliamentary sovereignty, constitutional statute

## Abstract

UK constitutional law establishes priority rules governing the relations among legal sources. According to the implied repeal rule, a later statute is preferred to and repeals an earlier statute where the two cannot stand together. There is a vast literature testing the rule’s application in future-facing scenarios: whether Parliament in enacting legislation is capable of legally binding its successors. This article instead adopts a backward-facing perspective, focusing on past enactments. I examine Parliament’s legislative power to disrupt how implied repeal applies to earlier, inconsistent statutes. This sheds light on Parliament’s capacity to shape the constitution’s architecture—here, by rearranging priority relations among existing statutes. I juxtapose the technique against the doctrine of constitutional statutes, and also address the implications for the doctrine of parliamentary sovereignty. Nor is the technique simply of academic interest. A backward-facing reprioritising regime has already been established in the legislation governing UK withdrawal from the EU. Lastly, the argument may be generalised to encompass other legislatures that also enjoy powers to disrupt the implied repeal rule normally operating among past statutes.

## 1. Introduction

In what ways can Parliament reshape the UK constitution? Legislation intervenes in the operation or composition of existing key organs of government,^1^ establishes and regulates new organs^2^ and changes the rights protections of subjects as against the state.^3^ In these ways, legislation as a source of constitutional legal norms is an ‘ordinary’ phenomenon,^4^ reinforcing how the constitution is developed through the ordinary legislative process.^5^

This article is about a different aspect of Parliament’s legislative powers that has constitutional significance: its power to influence the relations among existing statutes. UK constitutional law establishes priority rules governing the relations among legal sources. According to the rule on implied repeal, a later enactment is preferred to and repeals an earlier enactment where the two cannot stand together. There is a vast literature testing the rule’s application in future-facing scenarios: whether Parliament in enacting legislation is capable of legally binding its successors. This article instead adopts a backward-facing perspective, focusing on *past* enactments. I will examine Parliament’s legislative power to disrupt how implied repeal applies to earlier, inconsistent statutes. This power has yet to be analysed. It is operationally different from making new statutes or amending and repealing existing statutes. After situating the topic in light of existing thinking (section 2), I discuss two circumstances in which the technique may be used: Parliament’s decision to disrupt how implied repeal applies to earlier statutes based on its appraisal of their *content* (section 3); and reprioritisation that is sensitive to differing *procedures* by which statutes are enacted (section 4). I also (in section 3) juxtapose the technique against the doctrine of constitutional statutes, and address the implications for the doctrine of parliamentary sovereignty. The article wraps up with an assessment of the general practicability and desirability of such legislative reprioritisation (section 5).

The power to disrupt how implied repeal applies to existing statutory law goes to Parliament’s capacity to author a legislated constitution on its own terms. It invites us to consider how Parliament can direct the development of the constitution’s architecture, as compared, for instance, with judge-led processes. Nor is the issue merely of academic interest. As we shall see, it has been brought to life by legislation giving effect to the UK’s withdrawal from the EU, wherein an ambitious (backward-facing) reprioritising regime has been established. Lastly, while the UK, with its doctrine of parliamentary sovereignty, provides a useful case study of this power, the discussion also has broader relevance for legal systems with entrenched capital-C constitutions where the legislature may equally be empowered—through the ordinary legislative process—to modify the operation of implied repeal among past enactments.

## 2. Priorities

### A. King Rex the Law-Giver

To introduce the core issue, I will open with a fable about King Rex, who is the kingdom’s plenary legislator. The king is, of course, not a complex legislative body such as the UK Parliament, but the fable’s simplicity is useful in setting up the primary themes that I wish to examine involving priority relations among enactments and how these may be determined. The kingdom’s courts follow an implied repeal rule whereby they give effect to the latest enactment—as the most recent expression of King Rex’s will—and repeal earlier, inconsistent enactments. For a time, the arrangements suit successive monarchs and the courts. But the courts begin to reason that some enactments are distinctly important and warrant protection from being accidentally overridden by the latest kingly act. To meet this reasoning, the courts advance a new doctrine whereby certain acts satisfying the doctrine’s criteria of importance are overridable only if the king makes his intention to override sufficiently clear.

The doctrine takes hold. But the judiciary are not all agreed on its significance. For some the doctrine is innocuous, with the courts continuing to give effect to the king’s latest will albeit through a more sophisticated apparatus offering a measure of protection for the legislator’s own important historic (and still readily overridable) enactments. Other judges consider the developments more dramatic—in giving the courts scope to prioritise enactments and potentially even to review the contours of kingly law-making power.

In the court of King Rex, a rethink is also taking place. The king recognises that as legislator he has responsibility for curating the statute book. He is influenced by how his economic advisers describe states that are economically more *dirigiste* than *laissez-faire*, and seeks to leverage the insight in a constitutional setting with regard to his own law-making. So the king, not content to leave the matter solely to judges, intervenes from time to time by legislating to modify how implied repeal applies to earlier statutes. In this way, he prescriptively reprioritises existing statutory law—all in the service of ends that *he* adjudges important.

### B. Priority Relations in the Law

In reflecting on the circumstances facing modern legislative assemblies, I will draw *mutatis mutandis* on the allegory of King Rex—particularly its third act, involving legislative reprioritisation of past enactments through disruption of the implied repeal rule. I argue that such action may be pursued by modern legislative assemblies and, as I will explain shortly, UK legislative practice following withdrawal from the EU is already illustrative. Nonetheless, the possibilities and their constitutional implications have yet to be considered. I will examine two main sorts of legislative reprioritisation in sections 3 and 4 below. By way of groundwork, this section (i) unpacks the notion of priority relations in the law, (ii) discusses the priority rule of implied repeal and (iii) introduces the argument that Parliament may reprioritise the statute book by disrupting implied repeal’s application to past enactments.

Priority refers to the fact of preferring one thing to another; frequently, this is because the former is regarded as more important than the latter.^6^ Priority rules are, in HLA Hart’s terminology, secondary rules—ie rules about other rules. Some priority rules address the relations between (rules derived from different) sources—eg the preference in the UK for rules derived from Acts of Parliament over prerogative primary legislation,^7^ or the common law. In capital-C constitutions, certain priorities between sources may be formally expressed in the constitutional instrument. South Africa’s Constitution provides that it ‘is the supreme law of the Republic’,^8^ with further priority rules set out regarding national, provincial and municipal acts,^9^ and other sources such as international law.^10^ The operation of priority rules such as these is typically complex. In the UK, for instance, the relation between statute and common law may be burnished by rebuttable presumptions—eg while ‘an Act may abolish, modify or displace existing common law rules, expressly or by implication’, nonetheless ‘there remains a general presumption that the legislature does not intend to make changes to the common law’.^11^

Legal systems also characteristically manage preferences among rules derived from the same source, such as statute. In common with other jurisdictions, later statutory enactments in the UK are preferred to and repeal earlier, inconsistent enactments, and this rule on implied repeal is addressed next.

### C. Implied Repeal

Implied repeal is a priority rule. It is variously expressed as a rule according to which a later enactment has ‘priority’,^12^ takes ‘precedence’^13^ or ‘prevails’^14^ over an earlier, inconsistent enactment. Relevant case law, familiar to all law students, includes *Vauxhall Estates*^15^ and *Ellen Street Estates*.^16^

A more recent case, *Hamnett v Essex County Council*, offers a clear account: ‘where the provisions of two statutes cannot stand together, the later provisions prevail and the earlier provisions are treated as repealed by implication or amended to the extent necessary to remove the inconsistency’.^17^ This is ‘in accordance with the maxim *leges posteriors priores contrarias abrogant* (later laws abrogate earlier laws)’.^18^ Implied repeal and amendment are related—eg repeal of a provision may simultaneously be treated as amendment of the Act.^19^

Gross LJ in *Hamnett* helpfully provides a more complete statement of implied repeal by also identifying qualifications. First, the court ‘will not lightly invoke’ the rule on implied repeal,^20^ which operates only if the earlier enactment ‘is so inconsistent with or repugnant to [the later enactment] that the two are incapable of standing together’.^21^ The court will seek in the first instance to reconcile the two enactments through interpretative means. Second, the rule ‘is inapplicable or more difficult to apply where the earlier enactment is particular and the later general’.^22^ Here the court will strive to construe the earlier enactment as an exception to the later, more general regime before resorting to implied repeal.

Third, the implied repeal rule’s application to an earlier enactment is qualified if the enactment has constitutional status.^23^ This qualification is grounded in the doctrine of constitutional statutes. In the case of *Thoburn*, Laws LJ indicated that there are ‘now classes or types of legislative provision which cannot be repealed by mere implication’,^24^ and that repeal could only be effected ‘by express words in the later statute, or by words so specific that the inference … was irresistible’.^25^ Laws LJ explained that ‘a constitutional statute is one which (a) conditions the legal relationship between citizen and State in some general, overarching manner, or (b) enlarges or diminishes the scope of what we would now regard as fundamental constitutional rights’.^26^ It is also suggested that constitutional statutes are ‘more carefully protected’ from being modified by subordinate legislation.^27^

Lastly, priorities may operate among constitutional enactments themselves in the event of inconsistency, as indicated obiter dicta in the *HS2* case regarding the relation between article 9 of the Bill of Rights 1689 and the European Communities Act 1972.^28^

### D. Reprioritisation by Disrupting Implied Repeal

Parliament enjoys plenary powers to legislate on constitutionally significant subject matter—eg legislating to devolve power from Westminster to the UK’s nations, the establishment of a Supreme Court or human rights protections. But another sort of constitutionally significant intervention is available. Parliament may also disrupt the application of implied repeal to existing statutory law. The rule on implied repeal has constitutional significance both as a complex priority rule that manages relations between inconsistent requirements derived from the same source (ie statutes) and as a rule that supports parliamentary sovereignty (without prejudice to any preferred theoretical account)^29^ in that Parliament’s legislative powers extend to the repeal of earlier enactments either expressly or impliedly.

Throughout this article, when referring to legislative reprioritisation I mean (unless otherwise indicated) *legislative action that disrupts the operation of implied repeal among past enactments*. The focus is squarely on interference with implied repeal as a distinctive technique that warrants analysis. Through this technique, Parliament may enact legislation that prescriptively rearranges the priority relations among earlier statutes, or among classes of earlier statutes, regardless of their respective enactment dates.

## 3. Reprioritisation Based on Content

I will examine two main sorts of legislative reprioritisation: content-based reprioritisation, where Parliament decides to reassess the relative importance of earlier enactment *content* (section 3); and reprioritisation that is sensitive to differing *procedures* by which Acts of Parliament are enacted (section 4). Both involve Parliament disrupting the normal application of implied repeal among past enactments.

I will begin by considering statutes that are reprioritised given Parliament’s appraisal of the importance of their content. Several illustrative examples will be discussed: (i) potential reprioritisation to further protection of the environment; (ii) the wide-ranging scheme recently adopted under the European Union (Withdrawal) Act 2018; and (iii) a potential scheme involving parliamentary privilege and article 9 of the Bill of Rights 1689. The implications for our understanding of parliamentary sovereignty will also be addressed.

### A. The Environment

The following scenario involves a statutorily encoded prioritisation of certain environmental statutes:

***Statute A*** *—A 2035 Act of Parliament (Statute A) provides that statutes pertaining to state aid take effect subject to statutes that further greenhouse gas reduction and are to be construed or disapplied as appropriate.*

Suppose that by 2035 the general public is increasingly exercised about carbon emissions,^30^ with the dramatic observational effects of climate change—alongside ongoing anxiety about energy security and independence^31^—driving pressure for broader and tougher measures in the UK. International pressure also becomes acute following a confluence of severe climatic events that devastate vulnerable communities in developing and island states.^32^ The government seeks to move quickly. After EU withdrawal, the UK had (we may suppose) adopted a variety of bespoke legislative measures on state aid in order to boost selected industrial sectors. Without undoing these state aid measures through repeals, or attempting to rewrite them, the 2035 statute (Statute A) provides that legislation furthering the reduction of greenhouse gas emissions is preferred notwithstanding the existing legislation on state aid—the latter benefiting industry but at risk of impeding climate action. Litigation is likely under Statute A, with industry and environmental groups asking judges to resolve disputes about affected enactments. To illustrate the legal consequences, we may suppose that a 2029 enactment conferring state aid is found to take effect subject to a 2026 enactment furthering greenhouse gas reduction—and insofar as the 2029 Act cannot be construed compatibly, it is disapplied to extent of the inconsistency. The normal operation of the implied repeal rule is interrupted. This reordering of priority relations is repeated across the statute book and according to Parliament’s latest instruction, adopted in 2035: Statute A. In future, the government intends to revisit state aid legislation in order to ensure long-term sustainability, and plans to hold thorough consultation exercises involving industry, climate scientists, public health experts and other stakeholders.

The backward-facing operation on *pre*-2035 state aid legislation—eg a 2029 state aid enactment—has just been discussed. Future-facing ramifications for *post*-2035 state aid legislation may additionally be considered. Supposing that a 2040 statute on state aid is adopted, according to Statute A it must be construed compatibly with the 2026 greenhouse gas-reducing legislation. The interpretative obligation thus operates on all state aid legislation, whether adopted pre- or post-2035. But absent a *Factortame (No 2)*-type accommodation,^33^ future-facing *disapplication* of the 2040 state aid legislation is seemingly unavailable given the prevailing judicial approach to the doctrine of parliamentary sovereignty.

Statute A’s operation is constitutionally significant in reordering priority relations among past statutes and disrupting the constitutional expectation that under the implied repeal rule later-in-time statutes are preferred. The intervention is motivated in Statute A’s case by the practical challenge of rapidly achieving an objective—a reduction in emissions—that is considered *important*. It is also a systematic intervention of the sort that can only be established under an enacted scheme—it is infeasible for judges to achieve such reprioritisation in case law—although the courts are nonetheless substantially involved in administering the scheme. Reprioritising interventions may also be more localised as to subject matter—eg within the law of evidence, rebalancing earlier statutory rules on evidentiary presumptions to privilege one sort over another based on assessment of relative importance. Thus, the Uniform Rules of Evidence (1999) propose the following concerning US state law presumptions in civil proceedings:

Rule 302(b) Inconsistent presumptions. If presumptions are inconsistent, the presumption applies that is founded upon weightier considerations of policy. If considerations of policy are of equal weight, neither presumption applies.

These Rules, which are ‘approved and recommended for enactment in all states’,^34^ anticipate the reordering of past statutory presumptions—where these are inconsistent and regardless of dates of enactment—in accordance with the latest statutory instruction.

A subsidiary point may, in closing, be made about the scenario involving Statute A. The 2035 Act invites judicial discretion in that the courts must identify the enactments falling under its reprioritising scheme—eg which enactments ‘further greenhouse gas reduction’. Parliament may, of course, be more prescriptive:

Statute A* —A 2035 Act of Parliament (Statute A*) provides that the statutes listed in Schedule 1 take effect subject to the statutes listed in Schedule 2 and are to be construed or disapplied as appropriate.

Parliament exerts through Statute A* more fine-grained control of the reordering by naming affected enactments.

### B. UK Withdrawal from the EU

With Statutes A and A*, a picture is emerging of what legislative reprioritisation makes possible, but a recently adopted statute brings the issue to life. The European Union (Withdrawal) Act 2018, which gives effect to the UK’s withdrawal from the EU, signals how Parliament may legislate for a general rule disrupting implied repeal and thereby reordering priority relations among (classes of) earlier statutes in a wide-ranging and systematic manner.

Given UK absorption of EU law during 47 years of EU membership, the 2018 Act minimises post-withdrawal disruption by taking a snapshot of EU law on IP (‘implementation period’) completion day^35^ and converting it into domestic law as ‘retained EU law’. Under sections 3–4 of the Act, various EU law norms are now domesticated as retained EU law. Notably, section 2 of the Act *also* treats domestic primary (or subordinate) legislation that is ‘EU-derived’ as retained EU law^36^—eg relevant provisions of the Equality Act 2010.^37^ Finally, the 2018 Act maintains continuity with the previous state of affairs by domesticating the EU principle of supremacy under section 5(2), which now grants priority to relevant retained EU law over other pre-IP completion day domestic law.^38^ Legal continuity with the previous state of affairs during EU membership is thereby secured while leaving scope for future regulatory divergence.

A striking possibility emerges: the 2018 Act’s newly domesticated supremacy rule under section 5(2) may in theory reshape priority relations between existing classes of statutes. The thought here is that existing ‘EU-derived’ Acts of Parliament counting as retained EU law under section 2 may benefit from (backward-facing) domestic supremacy under section 5(2) and thereby take priority over other pre-IP completion day statutes regardless of respective enactment dates, indicating the disruption of implied repeal. The 2018 Act does not explicitly settle whether domestic Acts benefit from section 5(2), however, and the difficulty is that during UK membership of the EU the EU’s supremacy principle did not confer supremacy on Acts of Parliament but only on (qualifying) EU law norms. In understanding how the newly domesticated supremacy rule operates, much turns on the qualifying words ‘so far as relevant’ in section 5(2); in the government’s view, they indicate that domestic supremacy does not benefit domestic statutes falling under section 2 but only relevantly applies to those EU norms now domesticated under sections 3-4 and which *did* previously benefit from EU supremacy.^39^ While this is arguably the better view—the 2018 Act’s principal objective is to domesticate, not reshape, EU norms and doctrines—the question may require judicial clarification.^40^ It is nonetheless significant that the House of Lords Constitution Committee accepted that domestic statutes under section 2 could conceivably benefit from the section 5(2) domesticated supremacy rule; otherwise its criticism that the matter was unsettled would not be meaningful.^41^ Tellingly, the government’s response also offered no argument against the constitutional possibility of statutes under section 2 benefiting from domestic supremacy; the government position was only that the 2018 Act happened not to establish such a regime (in keeping with the previous state of affairs during EU membership).^42^

The upshot is that Parliament is legislatively empowered to establish a wide-ranging regime disrupting implied repeal’s application to (classes of) past enactments. In fact, it is arguable that the 2018 Act *already* establishes such a regime, albeit through its domestication of the EU doctrine of indirect effect rather than the supremacy principle. Simply stated, indirect effect requires that Member State authorities interpret domestic law in conformity with EU law. An EU law rule ‘is used as an aid to the interpretation of [a domestic] rule’,^43^ regardless of whether the EU rule meets the conditions for direct effect.^44^ EU indirect effect’s scope of operation encompasses cases of conflict between domestic and EU law.^45^ Addressing the conflict through interpretative means is preferred. Triggering the EU supremacy principle, leading to disapplication of the conflicting domestic norm, is a final option,^46^ and is in any event only available if the EU norm satisfies all conditions of EU direct effect (ie being sufficiently clear, precise and unconditional to supplant the offending domestic norm that faces disapplication).^47^

EU indirect effect’s operation is more forceful than it may first appear, however. Importantly, the EU Court of Justice insists that domestic authorities use EU indirect effect to resolve inconsistencies even in those clear-cut conflict scenarios where the EU supremacy principle *would* be triggered as a final option but happens nonetheless to be precluded for certain technical reasons. Notably, the EU supremacy principle may be unavailable because the EU norm, which may be sufficiently clear, precise and unconditional, falls foul of the bar on horizontal direct effect of EU directives. A well-known example is *Pfeiffer*, which concerned a conflict between provisions of an EU directive imposing a maximum weekly working time of 48 hours and German legislation providing for weekly working time in excess of 48 hours.^48^ The European Court of Justice (ECJ) found that the EU provision and the domestic rule were ‘not compatible’,^49^ recognised that the EU provision could in principle ‘produce direct effect’ because it was ‘unconditional and sufficiently precise’,^50^ but held that supremacy-based disapplication could not operate for a technical reason—namely, the restriction on horizontal direct effect of directives.^51^ The ECJ concluded:

In this instance, the principle of interpretation in conformity with Community law thus requires the referring court to do whatever lies within its jurisdiction, having regard to the whole body of rules of national law, to ensure that Directive 93/104 is fully effective, in order to prevent the maximum weekly working time laid down in Article 6(2) of the directive from being exceeded …^52^

The ECJ went on to stress that ‘*the national court must thus do whatever lies within its jurisdiction* to ensure that the maximum period of weekly working time, which is set at 48 hours by Article 6(2) of Directive 93/104, is not exceeded’.^53^

As with EU indirect effect, so with its domestic counterpart. Under the 2018 Act, the domesticated indirect effect doctrine addresses conflicts arising between retained EU law and other domestic law. For our purposes, domestic indirect effect operates to benefit certain pre-IP completion day statutes that are conflicting with other pre-IP completion day statutes in order to give full effect to relevant retained EU law—and, again, regardless of enactment date. The complex priority rule on implied repeal, with its various associated presumptions,^54^ may be disrupted for these purposes. This view is grounded in ECJ reasoning in cases such as *Pfeiffer*, where, as noted above, the domestic court must ‘do whatever lies within its jurisdiction, *having regard to the whole body of rules of national law*, to ensure that’ the relevant EU norm ‘is fully effective’.^55^ Specifically, indirect effect does not apply merely to domestic statutes ‘enacted in order to implement’ EU obligations but also ‘requires the national court to consider national law as a whole’—ie any domestic statute—in order to find an EU-compatible outcome.^56^ This impacts on the relations between statutes and the ECJ is explicit about this feature of indirect effect:

if … national law enables … *a provision of domestic law to be construed in such a way as to avoid conflict with another rule of domestic law or the scope of that provision to be restricted to that end by applying it only in so far as it is compatible with the rule concerned*, the national court is bound to use those methods in order to achieve the result sought by the directive.^57^

These remarks are reflective of ECJ case law, illustrating the scope and strength of EU indirect effect—and of its domesticated equivalent under the 2018 Act. The domestic indirect effect doctrine may accordingly intervene in a backward-facing way in conflict situations between older and more recent (pre-IP completion day) statutes—and insulate the older statute from being repealed or ‘restricted’—in the service of retained EU law. The consequence is a reordering of past (conflicting) statutes, disrupting the normal constitutional expectation under the implied repeal rule that the later-in-time statute takes effect in instances of inconsistency.

The distinctiveness of the 2018 Act thus lies in establishing a priority regime (through the domesticated indirect effect doctrine) that potentially disrupts implied repeal’s application to clear inconsistencies among past enactments. Furthermore, this wide-ranging intervention is a *directed* one—ie it is in the service of retained EU law, with the goal being to protect the integrity of the snapshot.^58^

### C. Constitutional Enactments

The constitutional significance of legislative reprioritisation is augmented if the past enactment content being reprioritised is itself material to the constitution’s operation. To help situate the discussion, I will focus on the doctrine of constitutional statutes, according to which certain enactments recognised under that doctrine receive protection from being impliedly repealed. Legislative reprioritisation may operate on such enactments as well. That is, Parliament may seek to reinforce or disrupt their priority relations *vis-à-vis* other statutes.

The circumstances by which constitutional statutes in the UK emerge (and, relatedly, the criteria for identifying them) remain under debate,^59^ and it is notable that some writers already seek to recognise the involvement of various constitutional actors, including Parliament. For Athanasios Psygkas, ‘the main objection to the current model of constitutional statutes is that it is a common law creature: the criteria and implications of designating statutes as constitutional were driven by the judiciary’.^60^ This underestimates other constitutional actors, Psygkas suggests, including Parliament. His argument draws on the debate in the United States about ‘super-statutes’,^61^ where it is suggested that constitutionally important commitments may emerge diffusely ‘through the more gradual process of legislation, administrative implementation, public feedback, and legislative reaffirmation and elaboration’.^62^ The ensuing super-statutes, such as the US Civil Rights Act of 1964, ‘successfully penetrate public normative and institutional culture in a deep way’^63^ and ‘tend to trump ordinary legislation when there are clashes or inconsistencies, even when principles of construction would suggest the opposite’.^64^ Drawing inspiration from this discussion, Psygkas proposes that our understanding of constitutional statutes should ‘move beyond the formalist criterion of judicial recognition’^65^ and look not just to a given statute’s content, but also its broader pre- and post-enactment history and the involvement of various constitutional actors.^66^ He is also careful to say that Parliament’s merely ‘labelling a statute’ as constitutional need not be determinative; equally, ‘a statute should not become constitutional just because a court has held so’.^67^

Identifying the facility afforded by legislative reprioritisation—prescriptive action by Parliament that is directed towards reprioritising constitutional enactments—intervenes in and supplements this debate. An illustration will ground the discussion and help also to reflect on possible motivating reasons for such action. Article 9 of the Bill of Rights 1689, concerning parliamentary privilege, is generally recognised as a constitutional enactment under the doctrine of constitutional statutes. In the *HS2* case, a query arose as to how to resolve ‘a conflict between [the] constitutional principle … embodied in article 9 of the Bill of Rights’ and the requirements of the (now repealed) European Communities Act 1972.^68^ Determining their relative priority was a matter ‘to be resolved by our courts’,^69^ the Supreme Court suggested in an earlier part of its judgment, while also presenting this in a later part as an implementation of Parliament’s decision.^70^ It may be noted that on questions about parliamentary privilege’s scope—what constitute privileged proceedings—*R v Chaytor* held that ‘the extent of parliamentary privilege is ultimately a matter for the court’, albeit ‘one on which the court will pay careful regard to any views expressed in Parliament’.^71^

Suppose that Parliament does intervene to reprioritise:

***Statute B*** *—A 2023 Act of Parliament (Statute B) provides that statutes take effect subject to article 9 of the Bill of Rights 1689 and are to be construed or disapplied as appropriate.*

This direction specifies the elevated enactment. Parliament seeks in Statute B to renew article 9’s importance in preventing judicial scrutiny of Parliament’s internal proceedings (which, let us suppose, are also more explicitly delimited). This helps secure a peaceful relationship between Parliament and the courts. Parliamentary law-makers cannot envision circumstances where article 9 should succumb to other statutory requirements—including where the latter are identified as constitutional under the doctrine of constitutional statutes—and do not wish to leave this matter to the courts given doubts about how they might proceed. Law-makers are aware that differences of opinion between judicial and legislative branches have materialised in other jurisdictions.^72^ Their motivations recall those of King Rex in our fable.^73^

A further reason motivates intervention by means of Statute B rather than, say, legislating anew with an up-to-date statement of the constitutional settlement. The Bill of Rights’ constitutional status is already established either under what is understood to be a judge-determined process or, as Psygkas suggests, through the involvement of various constitutional actors over a period of time. Given its role and venerability, the Bill of Rights has also come to exhibit an *expressive* value beyond its practical legal operation. Parliament would rightly be careful about pursuing amending or repealing legislation that dislodges an importantly embedded enactment, but rather may legislate to refine and improve its relations with other (important) enactments. In this vein, it may be noted that Australia^74^ and New Zealand^75^ have legislated to confirm article 9’s continuing application in their own systems, with more recent legislation being enacted that shapes and refines its operation.^76^

A final reason for a Statute B-type intervention is that it offers, in certain respects, more potent legal protection for the Bill of Rights than is available under the doctrine of constitutional statutes. Statute B’s reprioritising scheme, enacted in 2023, requires Bill of Rights-conforming interpretations of all statutes (whether pre- or post-2023); in addition, disapplication of *pre*-2023 statutes is at least available. By contrast, the protection afforded to the Bill of Rights by the doctrine of constitutional statutes is relatively thin gruel: insulation from implied repeal.^77^ The doctrine seems to have a (limited) defensive orientation.^78^ Importantly, the doctrine’s implications for interpretation remain uncertain—eg how far constitutional statutes are protected from being interpreted more narrowly to accommodate other enactments, and whether the doctrine might bring about circumstances in which constitutional statutes (*qua* constitutional) proactively motivate interpretations of other enactments. Clarification has yet to arrive in the case law or literature, which have mainly focused on a different question: whether a constitutional statute ‘might *itself* be subject to different techniques of interpretation from those normally applied to “ordinary” legislation’.^79^

It is thus uncertain how far the doctrine of constitutional statutes will insulate identified statutes from being interpretatively narrowed (as compared with insulation from repeal by implication). By contrast, a Statute B-type reprioritisation represents a prescriptive form of legislative intervention—involving disruption of implied repeal alongside explicit duties of interpretation and disapplication—that is difficult for courts to ignore. It reinforces how the technique provides a legally potent method for Parliament to protect and give effect to a constitutionally significant standard.

### D. Comparison

Legislative reprioritisation, in the way that I have discussed it, is an avant-garde technique. In the scenarios involving Statutes A, A* and B, a new Act of Parliament is prescriptively reorganising the priority relations among earlier statutes by disrupting the normal operation of implied repeal. While there is a legislative power to intervene in this way, thus far it has not clearly manifested—save for the example of the European Union (Withdrawal) Act 2018.

Another sort of backward-facing reprioritisation is more routine, however, with examples more readily found in legislative practice. In these instances, the relevant Act reprioritises its *own* requirements *vis-à-vis* those of an earlier enactment.^80^ For example, section 225C(8) of the Town and Country Planning Act 1990 provides:

This section has effect subject to—

(a) the other provisions of the enactments relating to town and country planning;
(b) the provisions of the enactments relating to historic buildings and ancient monuments; and
(c) Part 2 of the Food and Environmental Protection Act 1985 (which relates to deposits in the sea).

Other examples may be found in historic planning legislation—eg section 2(6)^81^ and section 29(8)^82^ of the Town and Country Planning Act 1962. Statutes also employ the expression ‘without prejudice’ (or analogues) to similar effect—eg section 36(2) of the Highways Act 1980, section 183(1) of the Water Resources Act 1991 and section 18(4) of the Civil Evidence Act 1968.^83^ Under section 56 of the Disability Discrimination Act 1995, a regime is established to help a potential claimant gather information from the potential respondent ahead of instituting proceedings, with qualifications stipulated under section 56(8):

This section is without prejudice to any other enactment or rule of law regulating interlocutory and preliminary matters in proceedings before a county court or industrial tribunal, and has effect subject to any enactment or rule of law regulating the admissibility of evidence in such proceedings.^84^

Similar techniques are adopted in other jurisdictions including international law.^85^

The aforementioned examples are reprioritising since they involve interruption of the otherwise applicable priority rule on implied repeal. The statutes apparently establish circumstances in which they should not be preferred, despite being enacted later in time, if inconsistent with earlier enactments. The motivating reason is also apparent: law-makers are aware that their latest intervention may overlap with and disturb the operation of important earlier rules, but also do not think it sensible to start afresh with a comprehensive new legislative framework accommodating additional variables. Hence, a reduced priority is ascribed to the latest (less important) enactment insofar as it intersects with (more important) requirements under earlier, inconsistent enactments.

A final comparative example is important. The now repealed European Communities Act 1972 provided under section 2(1) a conduit through which EU law took effect in the UK legal system. A priority rule was also established under section 2(4): ‘any enactment passed or to be passed … shall be construed and have effect subject to the foregoing provisions of this section’. Thus, according to section 2(4), any Act of Parliament, past or future, would operate subject to the requirements of section 2(1). For current purposes, the focus is on the section 2(4) priority rule’s *backward*-facing operation on past enactments.^86^ But here the legislated priority rule made no legal difference on the matter of priority.^87^ It subjected past (pre-1972) Acts to the requirements of the 1972 Act, as would be the case under the implied repeal rule.^88^ In addition, it is worth reiterating that, in its backward-facing operation, the 1972 Act’s section 2(4) priority rule operated to establish the priority over past enactments of its *own* requirements under section 2(1) (ie the conduit for EU law)—in keeping with the other comparative examples mentioned above.

Those earlier examples, such as the Town and Country Planning Act 1990, demonstrate how a statute can reprioritise its own content *vis-à-vis* past enactments. By contrast, the interventions involving Statutes A, A* and B—and under the European Union (Withdrawal) Act 2018—go one step further in establishing a rule reprioritising the relations *among* past enactments. It is not a distinction without difference. This latter sort of intervention is the apotheosis of legislative reprioritisation as a technique, and practical and theoretical implications follow. Practically speaking, it expands law-makers’ horizons in allowing for reprioritising schemes that are substantially more wide ranging and systematic—these being infeasible under statutory arrangements such as the Town and Country Planning Act 1990. Such a statute reprioritises certain of its *own* provisions *vis-à-vis* earlier statutes, and so in that way is operationally confined. But reprioritisation need not be so anchored, and a more liberated priority rule that governs relations *among* past statutes allows law-makers to establish ambitiously transformative schemes—as under Statute A or the European Union (Withdrawal) Act 2018.

Alongside the practical significance of such arrangements, there are theoretical implications as well. These go to our understanding of parliamentary sovereignty and are addressed next.

### E. Implications for Parliamentary Sovereignty

The constitutional possibility emerges of the statute book being punctuated by ‘reordering provisions’—legislative interventions akin to Statutes A or B that reorder priority relations among past enactments, regardless of their respective enactment dates and in the service of updated policy ends. It is important to note the permissibility of such reprioritisation under the doctrine of parliamentary sovereignty, including in its orthodox cast. In such circumstances, the courts would be asked to implement the latest relevant instruction of Parliament, directed here towards past enactments and interrupting implied repeal.

Drawing attention to this hitherto underappreciated power to reprioritise is helpful in revealing how the (future-facing) question whether Parliament can bind its successors, which is well rehearsed in the literature, can threaten to monopolise our thinking. We should remain alert to constitutionally significant ways in which Parliament may exert its legislative authority regarding existing statutes. The discussion also signals implications for how we understand parliamentary sovereignty. It challenges propositional inferences about implied repeal on which accounts of parliamentary sovereignty rely—and, in so doing, highlights and reaffirms the provisional character of our theorising on parliamentary sovereignty.

The linkage between accounts of parliamentary sovereignty and the rule on implied repeal is readily seen, for example, in the orthodox view of parliamentary sovereignty long associated with Dicey.^89^ Evidence for the orthodox view, it is said, lies in the fact that Parliament can expressly repeal any past enactment. The rule on implied repeal also supplies ‘strong evidence’.^90^ Alison Young, who offers a modern defence of the orthodox Diceyan view, uses the (pleasant) example of a 1995 statute requiring that parents of blue-eyed babies receive a £500 annual grant from the state, and a 2005 statute requiring a £750 annual grant:

The requirement that later statutes should be given precedence over earlier statutes upholds continuing parliamentary legislative supremacy. If, when faced with a conflict, courts were required to apply the provisions of the earlier statute, it would place a limit on the actions of future parliaments. To return to our example, if priority were given to the earlier statute, courts would have to apply the 1995 statute with its lower payment of £500. However, this would thwart the wishes of the later Parliament passing a statute in 2005 requiring a higher payment. In order to ensure that its wishes were applied, the later parliament would need to expressly repeal the provisions of the earlier statute. It would not be enough to merely pass legislation whose content repealed earlier provisions by implication—ie by containing provisions that contradicted earlier statutory enactments. The earlier parliament would have bound its future incarnations.^91^

Hence the importance of the implied repeal rule in ensuring that successors are not so bound; the 2005 statute takes priority and the 1995 statute is *pro tanto* repealed. The connection between implied repeal and the orthodox view is similarly presented by Nick Barber,^92^ Trevor Allan^93^ and William Wade, who, in sketching the Diceyan orthodoxy, refers to the ‘established rule about conflicting Acts of Parliament, namely that the later Act must prevail’.^94^ So too with Mark Elliott, who explains: ‘which act should take priority? The orthodox version of parliamentary sovereignty supplies a clear answer … when two acts conflict, the more recent … prevails.’^95^ Strictly speaking, the proposition suggested by these remarks is only contingently true on the orthodox view’s own terms. For, as we have seen, on the orthodox Diceyan understanding, Parliament is capable in its latest instruction of reordering earlier statutory relations by disrupting the backward-facing operation of implied repeal. In Young’s example, the priority relations between the 1995 and 2005 enactments may be so disrupted by a rule established under a 2023 statute.

Reflecting on legislative reprioritisation of the statute book sophisticates our thinking on parliamentary sovereignty. This includes not just the orthodox view, but other accounts which rest on propositions about the operation of implied repeal. According to a House of Commons Library Briefing Paper, ‘if two inconsistent Acts are passed at different times, the most recent must be enforced by the courts to the extent that they are inconsistent’.^96^ It is clear that such statements now require finessing.

## 4. Reprioritisation Based on Differing Legislative Procedures

Legislative reprioritisation may be content-driven, grounded in Parliament’s appraisal of enactment content. Parliament’s reprioritisation may also be sensitive to the differing legislative procedures that have been used—ie reprioritising past enactments in light of how they have been made. Two examples will be discussed alongside potential motivating reasons: (i) reprioritisation of legislation made using the ‘English Votes for English Laws’ (EVEL) procedure; and (ii) reprioritisation involving the Parliament Act 1911 procedure.

The first example concerns the experiment with EVEL in the Standing Orders of the House of Commons. EVEL was an internal legislative procedure introduced in response to the West Lothian question. As the issue has been framed, one consequence of the asymmetry of UK devolution is that MPs in English constituencies cannot (ordinarily) vote on matters devolved to the legislatures of Scotland, Wales and Northern Ireland, whereas MPs from the latter nations can (ordinarily) vote in the UK Parliament on bills affecting only England. Under EVEL, only MPs from English and at times Welsh constituencies would vote on bills or clauses affecting their constituents. EVEL was introduced in the Commons on 22 October 2015, suspended on 22 April 2020 and rescinded on 13 July 2021. It has been considered problematic in principle, and complex and time-consuming in practice. MPs from non-English nations were particularly critical of what they saw as an informal ‘two-tier’ hierarchy of MPs in the UK Parliament.

Another problem with EVEL has received less comment. Acts of the devolved legislatures remain subject to override by Acts of the UK Parliament (given parliamentary sovereignty). By contrast, EVEL produces its own legal imbalance since enactments adopted under EVEL are legally full-fledged Acts of the UK Parliament—as distinct from Acts of the devolved legislatures, which do not enjoy such legal status. EVEL generates its own asymmetry that is legally significant.

While the EVEL arrangements were flawed, it is possible nonetheless to imagine the UK Parliament legislating in response to the concern about imbalance. This may involve a generalised scheme regarding the subset of EVEL-based statutes:

***Statute C*** *—According to a 2016 Act of Parliament (Statute C) and unless otherwise provided, any statute adopted under EVEL takes effect subject to other statutes that are adopted under the standard legislative procedure. The 2016 Act’s application* ratione temporis *may be updated by ministerial regulations.*

Although Statute C does not eliminate the legal imbalance, it is a way for Parliament to recognise the sensitivity of the issue. It reduces the relative priority of EVEL-based statutes *vis-à-vis* other inconsistent statutes. The prospect of reprioritisation *downwards* was noted earlier.^97^ Possibilities such as these—seeking to refine the institutional response to the West Lothian question—become apparent once Parliament’s power to reshape priority relations among past enactments is recognised.

These points may be generalised. Insofar as the legislative reprioritisation of statutes is motivated by sensitivity towards the differing procedures used to enact them, this is another way for Parliament—or other relevant legislatures—to constitutionalise the statute book. To be clear, this differs from the use of special legislative procedures to amend a capital-C constitution.^98^ A note of caution is also needed. Leveraging variations in internal (ordinary) legislative procedures requires, at base, well-ordered procedures yet the state of affairs in many legislative assemblies is untidy. To illustrate, the use of legislative procedures in the UK Parliament is not well organised. According to *Erskine May*: ‘Particularly controversial clauses of … government bills (for example, those which raise questions of conscience) have been committed to a Committee of the whole House and the remaining clauses to a public bill committee’.^99^ But no invariable rule of practice has emerged; and a lack of consistency in the use of legislative procedures can also be seen with bills on important constitutional questions.^100^ Tidying up procedures would be a prerequisite for procedure-based reprioritisation.

A second example involves legislation made under the Parliament Act 1911 procedure whereby a public bill, notwithstanding rejection in the House of Lords, can be presented for Royal Assent and become an Act of Parliament. In this scenario, Parliament wishes to recognise the greater checks that Acts of Parliament have overcome if adopted with the consent of both Houses, and to express this by means of a reprioritising scheme:

***Statute D*** *—According to a 2023 Act of Parliament (Statute D) and unless otherwise provided, any statute made under the Parliament Act 1911 procedure without the consent of the House of Lords takes effect subject to other statutes adopted under the standard legislative procedure. The 2023 Act’s application* ratione temporis *may be updated by ministerial regulations.*

Statute D exemplifies reprioritisation that is reliant on an established and defined procedure—in comparison to internal procedures whose arrangements may (as just mentioned) be more malleable or disorganised.

In addition, the Statute D-type scenario casts further light on the constitutional possibilities thrown up by procedure-based reprioritisation. It is instructive here to juxtapose Statute D against the circumstances of *Jackson v Attorney-General*, where the court was invited to treat Acts made using the Parliament Act procedure as ‘delegated or subordinate, not primary’ legislation.^101^ The court declined, with judges repeatedly deprecating the claimants’ argument that these Acts were always (and would always be) of ‘derivative’ legal status^102^ and constituted a ‘form of sub-primary parliamentary legislation’.^103^ By contrast, Statute D makes no such status-based claims. It establishes an operational scheme governing relations among pre-2023 enactments, each of whose legal status remains undisturbed, and the scheme is, of course, revisable or reversible by Parliament. Finally, reflecting on a Statute D-type scheme reveals how sophisticated constitutional balances within legislative assemblies (bicameral or otherwise) can be calibrated and expressed not just through the design of the institutions or the design of their procedures, but also through the priority relations of enactments adopted under those (varying) procedures. Parliament as a constitutional actor can be prescriptive on institutional design (eg compositional reforms under the House of Lords Act 1999), regarding its internal procedures and, as Statute D indicates, in this third dimension as well.

## 5. Parliament’s Constitution

### A. What Is Possible

Legislative reprioritisation displaces the legal system’s normally applicable priority rule of implied repeal. It provides direction to the courts in reconciling inconsistencies arising among past enactments.^104^ The reprioritisation is adopted through, and vulnerable to change under, the ordinary legislative process.

Such directed intervention—legislative *dirigisme*, as in King Rex’s fable—presents a distinct constitutional opportunity. It permits a democratic legislature to pursue constitutionalising action in place of an entrenched capital-C constitution. The technique is, however, also subject to limitations. Notably, it is backward facing in that it operates on existing statutes. Moreover, it is constitutionalising action through the ordinary rather than a special legislative process, which sparks concern about process legitimacy.^105^ Of course, such worries arise already regarding ordinary legislation on constitutional matters generally—worries that are magnified by the gloomy condition of our (executive-dominated) parliamentary politics.

The emergent picture is of a complex statutory topography, with the relational importance of existing statutes shifting under various impulses including parliamentary direction. The UK supplies a useful case study in that pressures for constitutionalising action cannot be satisfied through amendments to a capital-C constitution. The argument also has relevance for legislatures in other jurisdictions which may pursue equivalent strategies under the umbrella of a capital-C constitution, particularly if this is difficult or practically impossible to amend.^106^ Furthermore, the argument enriches our general appreciation of how, beyond formal amendment rules in constitutional codes, constitutional change in legal systems may be effected variously through legislative, executive and judicial action.

It should be said that legislative reprioritisation, as a distinctive technique for influencing the system’s constitutional architecture, differs notably from another prospect—namely, law-makers taking notice of the doctrine of constitutional statutes and attempting to exploit it actively. Something close to that prospect has already been foreshadowed: ‘Draftsmen of new Acts will, of course, turn their minds to whether the courts are likely to construe them as constitutional enactments.’^107^ Two obvious differences may be highlighted. First, even if drafters seek actively to exploit the doctrine of constitutional statutes, it is a more limited form of agency (than in the case of deliberate reprioritisation), since a statute’s being identified as constitutional depends on other constitutional actors including, at minimum, judges.^108^ Second, on legal consequences, the doctrine offers a constitutional enactment a measure of insulation from implied repeal; this differs from a backward-facing reprioritising scheme, which prescriptively disrupts the operation of implied repeal among past enactments (of any sort).

Given the opportunity presented by legislative reprioritisation, how far will it be realised? Notwithstanding the European Union (Withdrawal) Act 2018, and looking ahead to how parliamentary practice may evolve, the answer depends on law-makers’ awareness of the facility and judgments as to what is practicable and desirable. The scenarios discussed earlier—eg Statutes A or B—were fashioned with these considerations in mind. For example, Statute A anticipates a scenario where the UK, subject to acute international and domestic pressure, seeks swift action to reduce emissions involving a reconfiguration of domestic (statutory) law. This pragmatic intervention is responsive to time-sensitive exigencies. As for Statute B, concerning article 9 of the Bill of Rights, the scenario evokes a legislature wishing to shape the UK’s constitutional architecture more prescriptively in the face of the doctrine of constitutional statutes and common law constitutionalism. The operational consequences of the latter developments may attract parliamentary scrutiny, with law-makers expressing disquiet about judges’ (perceived) shaping of the constitution.^109^ The situation recalls the fable of King Rex: he observes the kingdom’s judges moving to assess the relative importance of existing enactments and, rather than passively receiving such developments, he decides (as principal legislator) to cultivate the statute book’s priority relations in a legislatively more directed way.

### B. Hopes and Fears

The preceding discussion has examined ways in which Parliament is able (and liable) to reprioritise past enactments. As to how far Parliament should so act, the following discussion pursues some preliminary threads of inquiry about potential benefits and drawbacks.

For optimists, Parliament should strive to recalibrate the operation of the existing statute book to serve beneficent and up-to-date policy preferences—eg advancing a policy agenda favouring labour interests over trade liberalisation.^110^ Moreover, such legislative reprioritisation, since it operates on existing statutory law and is in that respect backward-facing, does not provoke the democratic worries associated with *future*-facing action. The prospect of Parliament legislating in order to bind successors—ie fettering future generations through reprioritisation effected through the ordinary legislative process—is standardly criticised for producing ‘most undesirable (and undemocratic) results’.^111^ A backward-facing intervention sidesteps such worries and indeed may be thought to fulfil contemporary democratic impulses.

A further advantage concerns Parliament’s managing of its relations with the courts. Preferences among rules, and among underpinning interests or values, are important in the law and frequently animate law-makers. It is generally the purview of the courts to construe statutes so that they interact harmoniously, but their judgments about the shape of the overall web may be interrogated. This could turn on the soundness of the evaluative choices being made by (unelected) judges, or perhaps because it is in the nature of successive case-by-case adjudication that wider elements of the (complex, interconnected and overlapping) structure are left untended or dissonant. Insofar as judicial decision-making is non-representative and piecemeal, there are considerations of process legitimacy that favour legislative action.^112^ On another perspective, Parliament should itself be taking greater command of the statute book’s complexity for which it holds primary responsibility. Governments of different political persuasions have pursued successive legislative agendas, leaving behind an assortment of enactments that may benefit from careful reordering—and as part of a broader arsenal of legislative techniques, including amendment, repeal and the introduction of legislated canons of construction. Parliament thus has reason to intervene to reorganise statutory relations. Limiting mishaps will depend on a sensibly crafted scheme. The more tightly defined and circumscribed it is—eg Statute A* rather than Statute A—the more straightforward it will be to achieve a predictable reordering. Conversely, a more loosely defined scheme, as in Statute A (‘exactly which statutes are furthering greenhouse gas reduction?’), will imply a preference for institutional co-determination by inviting judgment on the part of the courts.

This segues into advantages both epistemically and in respect of legal certainty. Legislative reprioritisation, as I have discussed it, has a backward-facing character in being addressed to past enactments. This operational limitation is in epistemic terms a strength. Law-makers may be expected to know the existing law and to make sound changes in light of their knowledge. Additionally, Parliament can rely on its intervention being given effect by the courts; they are duty-bound to apply the relational changes that Parliament has authored. This is a legally more certain means of reprioritising the statute book than techniques involving future-facing action—which run into obstacles posed by the judicial approach to parliamentary sovereignty and the fettering of successors. As for the doctrine of constitutional statutes, uncertainty surrounds whether a given enactment qualifies as constitutional, with the future-facing operational benefit limited to insulation from implied repeal by future enactments. Seeking legislatively to influence priority relations into the future is thus more fraught.^113^ Law-makers’ predictions are precarious, vulnerable to unanticipated developments and rightly contestable by later generations. This helps explain why, as a matter of political morality, we are discomforted by legislatures—even if well minded—freely binding their successors by means of enactments made through the ordinary legislative process. The contrast with backward-facing reprioritisation, even if this is significantly constitutionalising, is that Parliament’s actions are cabined and the implications more definite.

Legislative reprioritisation also presents some ominous signs. Naturally, there is worry that it will be used for unsound policy ends. For public lawyers, particular concern arises—again, on process legitimacy grounds—about reprioritisation being used to effect important *constitutional* change impacting on the operation of constitutional enactments or the constitution more generally. Since the technique is exercised through the ordinary legislative process, and in light of the (partisanised) state of parliamentary politics, there will rightly be anxiety about the constitutional levers that can be pulled. In the Westminster system, Parliament’s legislative agenda is normally government-dominated, so significant constitutional change by means of legislative reprioritisation is normally, and suboptimally, in ministerial hands. Critics may thus speak not of Parliament’s constitution, but the government’s. The concern furnishes reasons to favour reprioritising interventions—particularly on important constitutional matters—that hand to judges a greater institutional role in exercising interpretative judgment about the scheme’s operation, rather than a scheme that is legislatively highly prescriptive.^114^ It should also be reiterated that the interventions can be undone in the normal way by successive law-makers—hence the present government’s aim of revisiting the scheme under the European Union (Withdrawal) Act 2018.^115^

## 6. Concluding Remarks

Legislative reprioritisation is available to Parliament and has already been deployed. Its undoubtedly avant-garde character raises queries about how far it will be utilised in the future. The technique has constitutional ramifications in displacing the system’s priority rule on implied repeal that otherwise governs relations among past (inconsistent) enactments. It demonstrates how Parliament may prescriptively shape—disrupt—this aspect of the constitutional architecture, not merely remain a passive subject of it. It is also a significant way to influence the constitution directly through canonical (legislated) texts in circumstances where the system lacks a capital-C constitution.^116^ The technique’s constitutional significance is especially pronounced in scenarios where the enactment content being reprioritised—eg article 9 of the Bill of Rights—is itself material to the constitution’s operation.

Future-facing scenarios—whether and how far Parliament can legally bind its successors—tend to monopolise attention in the case law and literature. While those scenarios are undoubtedly important, the existing law matters too. It is the law that presently governs us and Parliament’s power to reprioritise its requirements should also be of interest—and for the reason that no serious controversy arises about the technique’s legal effectiveness.

Reflecting on avenues for future research, it may be beneficial to inspect further the implications of legislative reprioritisation for Parliament’s relationship with the courts. This brings us back, full circle, to the fable of King Rex, who *qua* legislator wishes to pursue greater ownership of the constitutional architecture immanent in the statute book—wherein enactments are already treated as constitutional by judges in their case law. Displacing background priority rules through prescriptive legislative intervention is a disruptive move, even if the scheme’s operation is backward-facing. If the legislature were more readily to intervene in this way—to pursue Parliament’s constitution—it is uncertain how judges would or should react. Legislators may argue in a high-minded (or high-handed) way that Parliament should proactively constitutionalise the statute book because it is better placed to do so than the judges, and perhaps in order to take the sting out of judicial restlessness underpinning such developments as ‘exceptional circumstances review’.^117^ In assessing Parliament’s activity, considerations of process and output legitimacy would come into play, and delicate balances among the branches of government would be implicated.

Much is already being written about judicial constitutionalism, the doctrine of constitutional statutes and important related developments. This article has sought to provide further illumination of what is possible through legislative powers. Its broader aim is to enrich the array of descriptive and evaluative questions about how constitutional law can and should be developed, and by whom.

## Footnotes

1 eg the Parliament Acts 1911 and 1949; the House of Lords Act 1999.

2 eg the Scotland Act 1998 establishing the Scottish Parliament and Government; the Constitutional Reform Act 2005, s 23 establishing a UK Supreme Court.

3 eg the Human Rights Act 1998, s 6.

4 Lael K Weis, ‘Environmental Constitutionalism: Aspiration or Transformation?’ (2018) 16 ICON 836, 869–70.

5 For a theoretical argument about the significance of ordinary legislation in constitution-making—which also proceeds on an openly instrumentalist footing, setting intrinsic (eg expressivist) considerations to one side—see Frank I Michelman, ‘What Do Constitutions Do that Statutes Don’t (Legally Speaking)?’ in Richard W Bauman and Tsvi Kahana (eds), *The Least Examined Branch: The Role of Legislatures in the Constitutional State* (CUP 2006) 273.

6 Priority may, of course, be grounded on a different measure—eg ordinal rankings in a dictionary where words beginning with the letter ‘a’ must come before those beginning with ‘b’.

7 UK Cabinet Manual (1st edn, Oct 2011) para 1.16: ‘An Order in Council made under the Royal Prerogative is regarded as a form of primary legislation. Examples of this are Orders for the Prorogation of Parliament.’

8 s 2.

9 eg s 44(4) on national legislation and s 156(3) on municipal by-laws.

10 eg s 232: ‘Customary international law is law in the Republic unless it is inconsistent with the Constitution or an Act of Parliament.’

11
*Bennion, Bailey and Norbury on Statutory Interpretation* (8th edn, LexisNexis 2020) s 25.6, also explaining: ‘The more fundamental the relevant common law rule or principle the stronger the presumption against legislative interference is likely to be’ (ibid). See also Daniel Greenberg, *Craies on Legislation* (12th edn, Sweet & Maxwell 2020) para 14.1.7.

12 eg TRS Allan, ‘Parliamentary Sovereignty: Lord Denning’s Dexterous Revolution’ (1983) 3 OJLS 22, 26 (‘takes effect in priority to previously existing law’); Pavlos Eleftheriadis, ‘Parliamentary Sovereignty and the Constitution’ (2009) 22 CJLJ 267, 279 (‘Wade has carefully explained … the priority of later statutes over earlier ones (i.e., the doctrine of implied repeal)’); NW Barber, ‘The Afterlife of Parliamentary Sovereignty’ (2011) 9 ICON 144, 151 (‘On both interpretations of Parliamentary sovereignty—Wade’s so-called orthodox version and Heuston’s manner-and-form variant—the House of Lords should have concluded that the conflict … resulted in the 1988 act taking priority, repealing the 1972 act so far as was necessary’).

13 eg Alison L Young, *Parliamentary Sovereignty and the Human Rights Act* (Hart Publishing 2009) 32: ‘The doctrine, simply stated, requires courts to give precedence to later statutes, should two statutes be found to conflict.’

14 eg JF Burrows, ‘Inconsistent Statutes’ (1976) 3 Otago Law Review 601, 611: ‘The doctrine of implied repeal embodies a rule of last resort: if all else fails, the second in time of two conflicting provisions prevails.’

15 [1932] 1 KB 733, 745–6 (Humphreys J), explaining that ‘where two inconsistent provisions are found in two Acts of Parliament, the one passed subsequently to the other, the later provision shall prevail and shall be deemed impliedly to repeal the earlier provision’.

16 [1934] 1 KB 590, 595–6 (Scrutton LJ) on express and implied repeal, describing Parliament either ‘repealing in terms the previous Act’ or ‘by enacting a provision which is clearly inconsistent with the previous Act’.

17 [2017] EWCA Civ 6, para 26 (Gross LJ).

18 ibid (quoting *Bennion* (n 11); emphasis added).

19 eg *Craies on Legislation* (n 11) paras 14.3.3–14.3.4 (‘When is an amendment a repeal?’); *Bennion* (n 11) s 8.4 on implied amendment. See also *Halsbury’s Laws of England: Statutes and Legislative Process*, vol 96 (Lexis 2018) paras 292–3 and indicating at para 292: ‘Amendment may take the form of, or include, repeal … If a provision of an Act is deleted, it can be said that the provision is “repealed” but that the Act is “amended”. In so far as an amendment also constitutes a repeal, the rules relating to repeals will apply.’

20
*Hamnett* (n 17) para 26.

21 ibid (quoting *Halsbury’s Laws of England*). See *Halsbury’s Laws of England: Statutes and Legislative Process* (n 19) paras 301–5; *Churchwardens and Overseers of West Ham v Fourth City Mutual Building Society* [1892] 1 QB 654, 658 (AL Smith).

22
*Hamnett* (n 17) para 26. See also *Bennion* (n 11) s 8.9 describing the ‘general presumption against repeal’ whose strength ‘varies according to the context’ and which is ‘particularly strong where general provision in an Act covers a situation for which specific provision is made in an earlier Act’; *Mary Seward v The Vera Cruz* (1884) 10 App Cas 59, 68 (Earl of Selborne LC).

23
*Hamnett* (n 17) para 26.

24
*Steve Thoburn v Sunderland City Council* [2002] EWHC 195 (Admin), para 60.

25 ibid para 63.

26 ibid para 62.

27
*Craies on Legislation* (n 11) para 1.5.3, describing greater protection afforded to constitutional statutes from ‘amendment under statutory powers expressly permitting amendment of legislation in general’. See also Tarunabh Khaitan, ‘“Constitution” as a Statutory Term’ (2013) 129 LQR 589, 598–601 on political assurances and statutory provisions constraining the use of delegated legislation, including Henry VIII powers, to make legislative changes of ‘constitutional significance’.

28
*R (HS2 Action Alliance) v Secretary of State for Transport* [2014] UKSC 3, paras 207–8 (Lords Neuberger and Mance).

29 The implied repeal rule has especial importance for those favouring the orthodox Diceyan view of parliamentary sovereignty—eg HWR Wade, ‘The Basis of Legal Sovereignty’ (1955) 13 CLJ 172, 174, suggesting that for an ‘orthodox’ lawyer ‘it is an invariable rule that in case of conflict between two Acts of Parliament, the later repeals the earlier’. See also Aileen Kavanagh, *Constitutional Review under the UK Human Rights Act* (CUP 2009) 297: ‘The doctrine of implied repeal is widely thought to be a corollary of the principle of parliamentary sovereignty, because it is a way of ensuring that no Parliament can bind a future Parliament, as the orthodox Diceyan account of parliamentary sovereignty requires.’ I return to parliamentary sovereignty in s 3E below.

30 See eg the Assessment Reports of the Intergovernmental Panel on Climate Change (IPCC) <https://www.ipcc.ch/> (last accessed 13 February 2023).

31 eg HM Government, ‘British Energy Security Strategy: Secure, Clean and Affordable British Energy for the Long Term’ (April 2022).

32 The IPCC’s 2022 Assessment Report (n 30) describes regions that have ‘high vulnerability to climatic hazard’—eg in East, Central and West Africa and Small Island Developing States—in H-O Pörtner et al (eds), *Climate Change 2022: Impacts, Adaptation and Vulnerability. Contribution of Working Group II to the Sixth Assessment Report of the Intergovernmental Panel on Climate Change* (CUP 2022) 12.

33
*R v Secretary of State for Transport, ex parte Factortame Ltd (No 2)* [1991] 1 AC 603.

34 ‘Uniform Rules of Evidence Act’ (1999) 5 <www.uniformlaws.org> (last accessed 13 February 2023).

35 The European Union (Withdrawal Agreement) Act 2020, s 39(2) defines IP completion day as ‘11.00 p.m. on 31 December 2020’.

36 Its classification as ‘retained EU law’ notwithstanding, ss 7(1)–(1A) confirm that s 2 domestic legislation retains its domestic legal status as primary or subordinate legislation.

37 This Act consolidates various domestic enactments giving effect to EU law on discrimination and equality.

38 s 5(2) provides that ‘the principle of the supremacy of EU law continues to apply on or after IP completion day so far as relevant to the interpretation, disapplication or quashing of any enactment or rule of law passed or made before IP completion day’.

39 House of Lords Constitution Committee, ‘European Union (Withdrawal) Bill’ (29 January 2018) HL 69, para 82: ‘The Solicitor General appears to take the view that retained EU law will benefit from the “supremacy principle” (in respect of pre-exit domestic law) only if it corresponds to pre-exit EU law that itself benefitted from the “supremacy principle”.’

40 The Constitution Committee was critical of the ‘ambiguous’ situation; see ibid para 83. A contrary view rejecting any ambiguity is taken by the Bingham Centre for the Rule of Law, ‘The EU (Withdrawal) Bill: A Rule of Law Analysis of Clauses 1–6’ (21 February 2018) para 107. See further Asif Hameed, ‘UK Withdrawal from the EU: Supremacy, Indirect Effect and Retained EU Law’ (2022) 85 MLR 726, 731–2.

41 Constitution Committee, ‘European Union (Withdrawal) Bill’ (n 39) para 83.

42 ibid para 82.

43 Gerrit Betlem, ‘The Doctrine of Consistent Interpretation—Managing Legal Uncertainty’ (2002) 22 OJLS 397.

44 eg Case 14/83 *Von Colson* EU:C:1984:153, paras 27–8.

45 eg Betlem (n 43) 399.

46 eg Case C-282/10 *Dominguez* EU:C:2012:33, para 23.

47 Case C-573/17 *Popławski* EU:C:2019:530, paras 60–2.

48 Joined Cases C-397/01 to C-403/01 *Pfeiffer* EU:C:2004:584, para 2.

49 ibid paras 100–2.

50 ibid paras 103–6.

51 ibid para 109.

52 ibid para 118.

53 ibid para 119 (emphasis added).

54 s 2C above.

55
*Pfeiffer* (n 48) para 118 (emphasis added).

56 ibid para 115.

57 ibid para 116 (emphasis added), reaffirmed in eg Case C-12/08 *Mono Car Styling SA* EU:C:2009:466, para 63.

58 The ways in which the 2018 Act domesticates the indirect effect doctrine are discussed further in Hameed (n 40) 744–54.

59 eg Farrah Ahmed and Adam Perry, ‘Constitutional Statutes’ (2017) 37 OJLS 461. See also Richard Albert and Joel I Colón-Ríos (eds), *Quasi-Constitutionality and Constitutional Statutes: Forms, Functions, Applications* (Routledge 2019), where the doctrine and its analogues are explored across a range of jurisdictions.

60 Athanasios Psygkas, ‘The United Kingdom’s Statutory Constitution’ (2020) 40 OJLS 449, 456.

61 eg William N Eskridge Jr and John Ferejohn, ‘Super-Statutes’ (2001) 50 Duke LJ 1215, 1216: ‘A super-statute is a law or series of laws that (1) seeks to establish a new normative or institutional framework for state policy and (2) over time does “stick” in the public culture such that (3) the super-statute and its institutional or normative principles have a broad effect on the law—including an effect beyond the four corners of the statute.’ See also William N Eskridge Jr and John Ferejohn, *A Republic of Statutes: The New American Constitution* (Yale UP 2010). Interest has grown also in such notions as ‘super-precedents’ in constitutional law—eg Michael J Gerhardt, ‘Super Precedent’ (2006) 90 Minn L Rev 1204.

62 Eskridge and Ferejohn, *A Republic of Statutes* (n 61) 14.

63 Eskridge and Ferejohn, ‘Super-Statutes’ (n 61) 1215.

64 ibid 1216.

65 Psygkas (n 60) 456.

66 ibid 458.

67 ibid 456.

68
*HS2* (n 28) para 79. Article 9 provides: ‘That the Freedome of Speech and Debates or Proceedings in Parlyament ought not to be impeached or questioned in any Court or Place out of Parlyament.’ In *R v Chaytor* [2010] UKSC 52, paras 12–13 it was clarified that the claim to privilege may rely on two bases: a statutory basis under art 9 and a common law basis known as the exclusive cognisance of Parliament ‘that has its origin before 1689 and which is wider than, and embraces, article 9’ (Lord Phillips).

69
*HS2* (n 28) para 79: ‘If there is a conflict between a constitutional principle, such as that embodied in article 9 of the Bill of Rights, and EU law, that conflict has to be resolved by our courts as an issue arising under the constitutional law of the United Kingdom.’

70 ibid para 208: ‘It is, putting the point at its lowest, certainly arguable (and it is for United Kingdom law and courts to determine) that there may be fundamental principles, whether contained in other constitutional instruments or recognised at common law, of which *Parliament* when it enacted the European Communities Act 1972 did not either contemplate or authorise the abrogation’ (emphasis added).

71 [2010] UKSC 52, para 16 (Lord Phillips). See also ibid para 15 explaining that this ‘applies as much to … an issue as to the scope of the exclusive cognisance of Parliament as it does to an issue as to the application of article 9’.

72 In New Zealand, the Supreme Court’s definition of proceedings in Parliament in *Attorney-General v Leigh* [2011] NZSC 106 was deemed problematic, and subsequently nullified by the Parliamentary Privilege Act 2014 under s 10.

73 s 2A above.

74 See the Parliamentary Privileges Act 1987, s 16.

75 See the Imperial Laws Application Act 1988, s 3(1) and Sch 1 (where it is listed as a ‘constitutional enactment’).

76 In New Zealand, see eg the Parliamentary Privilege Act 2014, s 9: ‘Article 9 of the Bill of Rights 1688 must be taken to have, in addition to any other operation, the effect required by this subpart.’

77
*Thoburn* (n 24) para 63. On the possibility of repeal by necessary implication where the inference is irresistible, see Farrah Ahmed and Adam Perry, ‘The Quasi-Entrenchment of Constitutional Statutes’ (2014) 73 CLJ 514. There is also case law suggesting that constitutional statutes, or possibly some of them, cannot be impliedly repealed at all—eg *H v Lord Advocate* [2012] UKSC 24, para 30, where Lord Hope indicated that ‘only an express provision’ could override the Scotland Act 1998 ‘because of the fundamental constitutional nature of the settlement that was achieved by the Scotland Act. This in itself must be held to render it incapable of being altered otherwise than by an express enactment’.

78 eg *Craies on Legislation* (n 11) para 1.5.3: ‘While the nature of constitutional enactments is that they are more protected from interference by other legislation, they have no greater strength than other enactments when it comes to themselves interfering with other legislation or law’ (footnote omitted).

79 David Feldman, ‘The Nature and Significance of “Constitutional” Legislation’ (2013) 129 LQR 343, 355 (emphasis added). See also David Feldman, ‘Statutory Interpretation and Constitutional Legislation’ (2014) 130 LQR 473 (taking the view, in summary, that ‘the techniques of interpretation appropriate for constitutional legislation are essentially the same as those used for any legislation, although the weights given to them may be different when the text and its interpreters have special roles’); Khaitan (n 27) 593–7. Regarding case law, see eg *Robinson v Secretary of State for Northern Ireland* [2002] UKHL 32, para 11, indicating that the Northern Ireland Act 1998 should ‘be interpreted generously and purposively, bearing in mind the values which the constitutional provisions are intended to embody’ (Lord Bingham). More recent case law seemingly rejects this approach—eg *Imperial Tobacco Ltd v Lord Advocate* [2012] UKSC 61, paras 14–15, indicating that the relevant rules under the Scotland Act 1998 ‘must be interpreted in the same way as any other rules that are found in a UK statute’ and that ‘the description of the Act as a constitutional statute cannot be taken, in itself, to be a guide to its interpretation. The statute must be interpreted like any other statute’ (Lord Hope). *Imperial Tobacco* confirms the approach taken a month earlier regarding the Government of Wales Act 2006 in *Attorney General v National Assembly for Wales Commission* [2012] UKSC 53, para 80 (Lord Hope). A contrary view of the developing case law is indicated in eg Stephen J Dimelow, ‘The Interpretation of “Constitutional” Statutes’ (2013) 129 LQR 498, 502, suggesting that the case law may alternatively be seen ‘as a clarification of the decision in *Robinson*’.

80 It should be added that purely *intra*-statutory arrangements are not a relevant kind of reprioritisation for current purposes since they do not implicate the system’s background priority rules regarding relations between statutes. An example of an intra-statutory arrangement is the Corporation Tax Act 2009, s 51(1): ‘Any relevant permissive rule in this Part … has priority over any relevant prohibitive rule [in the same Part].’

81 Subjecting relevant provisions of the 1962 Act to the National Parks and Access to the Countryside Act 1949, s 8 (now repealed).

82 ‘The preceding provisions of this section shall have effect subject to [the stipulated] provisions’ of the Forestry Act 1951 and Opencast Coal Act 1958.

83 ‘Nothing in this Act shall prejudice the operation of any enactment which provides (in whatever words) that any answer or evidence given by a person in specified circumstances shall not be admissible in evidence against him or some other person in any proceedings or class of proceedings (however described).’

84 The Disability Discrimination Act 1995 currently applies to Northern Ireland only; in other UK nations, it has been replaced by the Equality Act 2010.

85 States are encouraged to draft treaties that make specific provision—‘conflict clauses’—for their relations with other treaties, a practice that is supported under the 1969 Vienna Convention on the Law of Treaties, art 30(2). See also eg ‘Conclusions of the Work of the Study Group on the Fragmentation of International Law: Difficulties Arising from the Diversification and Expansion of International Law’ (2006) II(2) Yearbook of the International Law Commission 177, para 30: ‘When States enter into a treaty that might conflict with other treaties, they should aim to settle the relationship between such treaties by adopting appropriate conflict clauses.’ Examples include the Charter of the United Nations, art 103; the United States-Mexico-Canada Agreement, arts 1.3 and 32.3 (replacing the North American Free Trade Agreement); the 1982 United Nations Convention on the Law of the Sea, art 237; and the 1992 Convention on Biological Diversity, art 22.

86 I leave to one side the s 2(4) priority rule’s future-facing application to future Acts; the issue was litigated in *Factortame (No 2)* (n 33) and its implications for parliamentary sovereignty have been exhaustively scrutinised.

87 One unrelated operational difference is that earlier enactments were now to be disapplied if inconsistent with s 2(1) requirements rather than repealed. The 1972 Act, under ss 2(1) and (4), thus apparently established a regime that within its (backward-facing) scope of operation disabled implied repeal. This is not, however, an unusual intervention. Legislative schemes providing for limited disapplication of the requirements of past statutes have appeared prior to the adoption of the 1972 Act—eg the Housing Act 1957, Sch 1, para 1(2)—and subsequently—eg the Anti-social Behaviour, Crime and Policing Act 2014, s 17 or the High Speed Rail (London–West Midlands) Act 2017, s 38.

88 Analogous statutory arrangements are not uncommon—see eg Burrows (n 14) 612, discussing ‘the not uncommon statutory provision that a certain statute is to prevail “notwithstanding anything in any other act”’ and acknowledging that this is ‘probably only effective with regard to *earlier* inconsistent acts’ (original emphasis; footnote omitted).

89 AV Dicey, *Introduction to the Study of the Law of the Constitution* (10th edn, Macmillan 1959) 39–40.

90 Young (n 13) 32.

91 ibid 33.

92 Barber (n 12) 145: ‘when there is conflict between an older and a newer statute, [its] resolution … must give legal force to the newer statute—a resolution which may require a court to find that elements of the earlier statute are impliedly repealed’ (footnotes omitted).

93 Allan (n 12) 25: ‘The essence of the traditional doctrine of parliamentary sovereignty is that the courts should give effect to the most recent expression of parliamentary intent: the rule protecting implied repeal is simply one consequence of that doctrine.’

94 HWR Wade, ‘Sovereignty—Revolution or Evolution?’ (1996) 112 LQR 568.

95 Mark Elliott, ‘United Kingdom: Parliamentary Sovereignty under Pressure’ (2004) 2 ICON 545, 550. See also Mark Elliott, ‘Embracing “Constitutional” Legislation: Towards Fundamental Law?’ (2003) 54 NILQ 25: ‘The notion that Parliament’s legislative supremacy is ongoing or continuing implies that it is the courts’ duty to give effect to the most recent expression of Parliament’s will. Consequently, if two pieces of legislation conflict with one another, the courts are obliged to give effect to the more recent expression of Parliament’s intention’ (footnotes omitted).

96 Graeme Cowie, ‘The status of “retained EU law”’ (30 July 2019) House of Commons Library Briefing Paper No 08375, 9 (footnote omitted).

97 Several examples involving statutes reprioritising their own content downwards—eg the Town and Country Planning Act 1990 or the Disability Discrimination Act 1995—were discussed in s 3D above.

98 See eg the procedure set out in the Australian Constitution, s 128. As explained in DR Elder and PE Fowler (eds), *House of Representatives Practice* (7th edn, 2018) ch 10 (‘Procedural variations for different categories of bills’) 385: ‘The passage of a bill proposing to alter the Constitution is the same as for an ordinary bill, with the exception that the third reading must be agreed to by an absolute majority’ in each House.

99
*Erskine May: Parliamentary Practice* (25th edn, LexisNexis 2019) para 28.83 (footnote omitted). Examples include the Human Fertilisation and Embryology Bill [Lords] (1989–90) and the Criminal Justice Bill (1990–91).

100 ibid para 28.63: ‘It is common practice for government bills of “first-class” constitutional importance to be committed to a Committee of the whole House’ (footnote omitted). Examples include the European Communities Bill (1971–1972) and the European Union (Withdrawal) Bill (2017–2019)—ibid para 28.83. But equally ‘there is no invariable rule to that effect’ nor a ‘settled definition’ of such bills, and ‘Less significant bills may also be committed to a Committee of the whole House’—ibid para 28.63. These include ‘emergency and other expedited legislation’, such as the Banking (Special Provisions) Bill (2007–08), and ‘bills of a very uncontroversial nature such as Consolidation bills’—ibid para 28.83 (footnotes omitted). See also Robert Hazell, ‘Time for a New Convention: Parliamentary Scrutiny of Constitutional Bills 1997–2005’ [2006] PL 247.

101 [2005] UKHL 56, para 7 (Lord Bingham).

102 ibid para 22 (Lord Bingham).

103 ibid para 24 (Lord Bingham), para 94 (Lord Nicholls).

104 How directed the intervention is may vary, as demonstrated, for instance, in the contrast between Statutes A and A* discussed in s 3 above.

105 See further s 5B below discussing benefits and drawbacks.

106 Although the elderly US Constitution has been formally amended 27 times under art V, often in turbulent conditions, amendment is now widely thought impracticable given modern political polarisation. Nonetheless, as has been noted in Eskridge and Ferejohn, *A Republic of Statutes* (n 61) 5: ‘the inflexible Constitution has not stopped Americans from undertaking fundamental commitments, creating new rights, abolishing outrageous injustices. The very obduracy of the Constitution has channeled Americans towards the republic of statutes … Americans have created statutory and administrative rights and liberties that are both less dramatic and much broader than Large “C” Constitutional rights’.

107
*Craies on Legislation* (n 11) para 1.5.3.

108 See s 2B above.

109 The latter anxiety is reflected in the output of groups such as the Judicial Power Project in the UK <https://judicialpowerproject.org.uk> (last accessed 13 February 2023) and in work on other jurisdictions—eg Ran Hirschl, *Torwards Juristocracy: The Origins and Consequences of the New Constitutionalism* (Harvard UP 2004); Mark Tushnet, *Taking the Constitution Away from the Courts* (Princeton UP 1999); Mark Tushnet, *Taking Back the Constitution: Activist Judges and the Next Age of American Law* (Yale UP 2020).

110 The literature examining interactions between the two is, of course, vast. Some main contours are sketched in Christopher McCrudden and Anne Davies, ‘A Perspective on Trade and Labour Rights’ in Francesco Francioni (ed), *Environment, Human Rights and International Trade* (Hart Publishing 2001) 179, 183–8.

111 Allan (n 12) 28 (footnote omitted).

112 Analogous concerns may be found in eg Jeff King, ‘The Democratic Case for a Written Constitution’ (2019) 72 CLP 1, 19, raising a worry about judicial action under the doctrine of constitutional statutes: ‘the move being made here is very significant constitutionally. Authority for the new doctrine should issue from Parliament … or from the people directly under a written constitution, rather than through *ad hoc* recognition under the common law.’

113 While judicial institutions tend to benefit from being presented with actual—including novel and hitherto unforeseen—fact situations instantiated by the parties before them, some courts may exercise ‘abstract review’ on constitutional questions. Such review requires anticipation of the future and also presents epistemic challenges. See eg the German Federal Constitutional Court’s powers of abstract review under art 93(1) of the Basic Law (and, on the applicable procedure, ss 76–9 of the Federal Constitutional Court Act).

114 In a similar vein, see eg Alison L Young, ‘Constitutional Methodology and Brexit: Adopting a Model-Theoretic Approach’ in Dimitrios Kyritsis and Stuart Lakin (eds), *The Methodology of Constitutional Theory* (Hart Publishing 2022) 71, 95: ‘the power to create or modify fundamental aspects of the Constitution should not belong solely to the Westminster legislature … both the courts and the legislature have distinct strengths and weaknesses when determining constitutional issues given their distinct composition, expertise, and their decision-making processes … Parliamentary legislative supremacy is adhered to in order to ensure a more justifiable means of modifying the UK Constitution … Without it, Parliament alone would be able to entrench legislation, thereby modifying the Constitution. My theory argues that modifications of the Constitution are more legitimate and more likely to promote valuable outcomes if they involve both the legislature and the courts.’

115 A flavour of the government’s concerns can be found in the background briefing notes to the proposed Brexit Freedoms Bill in the 2022 Queen’s Speech: ‘The supremacy of EU law still applies to legislation passed before we left the EU; a total of 2,376 Acts of Parliament. By removing the principle of supremacy, the relationship between EU-derived law and those Acts of Parliament will be changed to ensure that EU-derived law no longer takes priority over laws passed by the UK Parliament. The Bill is an opportunity to … ensure that there is no higher law than an Act of Parliament’ (10 May 2022) <https://www.gov.uk/government/publications/queens-speech-2022-background-briefing-notes> (last accessed 13 February 2023). The government has now proceeded with the Retained EU Law (Revocation and Reform) Bill 2022–23, which is currently being scrutinised in Parliament.

116 The technique is also available to equivalently empowered legislatures operating under capital-C constitutions, and may be exploited particularly where the constitution is difficult or practically impossible to amend.

117 See Hayley J Hooper, ‘Legality, Legitimacy, and Legislation: The Role of Exceptional Circumstances in Common Law Judicial Review’ (2021) 41 OJLS 142, discussing *Jackson v Attorney General* and subsequent case law. This includes *Moohan v Lord Advocate* [2014] UKSC 67, para 35, addressing the prospects of review where ‘a parliamentary majority abusively sought to entrench its power by a curtailment of the franchise’ (Lord Hodge).

## Notes

I am very grateful to the anonymous reviewers and the General Editor, Liz Fisher, for their detailed and valuable feedback.

